# A Bit-Encoding Based New Data Structure for Time and Memory Efficient Handling of Spike Times in an Electrophysiological Setup

**DOI:** 10.1007/s12021-018-9367-z

**Published:** 2018-03-05

**Authors:** Bengt Ljungquist, Per Petersson, Anders J. Johansson, Jens Schouenborg, Martin Garwicz

**Affiliations:** 1 Neuronano Research Center, Integrative Neurophysiology and Neurotechnology, Lund University, Scheelevägen 2, 223 81 Lund, Sweden; 2 Department of Integrative Medical Biology, Umeå University, Linnéus väg 9, 901 87 Umeå, Sweden

**Keywords:** Electrophysiology, Databases, Data encoding, Real time

## Abstract

Recent neuroscientific and technical developments of brain machine interfaces have put increasing demands on neuroinformatic databases and data handling software, especially when managing data in real time from large numbers of neurons. Extrapolating these developments we here set out to construct a scalable software architecture that would enable near-future massive parallel recording, organization and analysis of neurophysiological data on a standard computer. To this end we combined, for the first time in the present context, bit-encoding of spike data with a specific communication format for real time transfer and storage of neuronal data, synchronized by a common time base across all unit sources. We demonstrate that our architecture can simultaneously handle data from more than one million neurons and provide, in real time (< 25 ms), feedback based on analysis of previously recorded data. In addition to managing recordings from very large numbers of neurons in real time, it also has the capacity to handle the extensive periods of recording time necessary in certain scientific and clinical applications. Furthermore, the bit-encoding proposed has the additional advantage of allowing an extremely fast analysis of spatiotemporal spike patterns in a large number of neurons. Thus, we conclude that this architecture is well suited to support current and near-future Brain Machine Interface requirements.

## Introduction

Brain Machine Interfaces (BMI) and Brain Computer Interfaces (BCI) have developed substantially during the past decades. Within the field of neural prosthetics, for instance, the general feasibility of real time control of robotic arms using multi-electrode-array recordings of cortical neural activity has been demonstrated (Wessberg et al. 2000) and, more recently, a robotic device allowing advanced arm and hand movements has been successfully implemented in tetraplegic subjects (Velliste et al. 2008; Collinger et al. 2013; Hochberg et al. 2012; Gilja et al. 2012). This development has depended partly on the identification of important principles of motor control, revealed by neurophysiological investigations of neural activity in awake, behaving animals (Monfils et al. 2005), and partly on advances within the field of robotics (Velliste et al. 2008). However, a functional interaction between the brain and robotic devices or computers also requires sophisticated neuroinformatics to ensure an efficient organization and analysis of neural data.

The demands on hardware and software used in the context of BMI/BCI are already high, as recent studies have used recordings of up to 1792 channels for a single subject (Schwarz et al. 2014). However, demands can be foreseen to further increase in the near future, as BMI and BCI reach new levels of sophistication, for example by including real time feedback protocols. Furthermore, in January 2016 DARPA (U.S. Defense Advanced Research Project Agency) announced the Neural Engineering System Design (NESD) program (Miranda et al. 2015), in which it aims for fully implantable devices able to record from up to one million neurons and stimulate 100 000 neurons, supporting a bidirectional BMI. Although the hardware part to perform this feat is a challenge in itself, the neuroinformatic requirements are immense, surpassing those previously implemented in currently available neuroinformatics systems. Unless adequately addressed, this may potentially become the rate limiting factor for the development of this field of neurotechnology in the near future.

In a previous paper (Ljungquist et al. 2011), we showed how a traditional Relational DataBase Managements System (RDBMS), used for storing neural data, might be combined with a data transfer mechanism. RDBMSs have also been used by other groups to structure and store electrophysiological data (Rautenberg et al. 2011). However, as RDBMSs are not built for large data sets, they scale poorly with binary data and multidimensional time series data such as electrophysiological and neuroimaging data. Hierarchical Data Format (HDF5) https://www.hdfgroup.org, by contrast, is a data format that potentially fulfills the requirements outlined above and has received increasing attention within the biomedical community for storing data (Dougherty et al. 2009; Mouček et al. 2014; Chintaluri et al. 2014). It supports a large variety of data types, and is designed for flexible, efficient and parallel I/O also for complex data, including high dimensional data. Here we take advantage of the HDF5 file format support for high parallel throughput and introduce bit-encoding to allow compression of large amounts of data. Although progress has been made recently (Rossant et al. 2016), spike sorting still remains a challenge. In this work, we assume that a correct online spike sorting and tracking is performed by each recording system. We also assume that the spike sorting results in single units being identified.

Current initiatives to build open source hardware as well as software for electrophysiological recording and analysis, are using both HDF5 formats as well as custom formats to store data, for example Open Ephys http://www.open-ephys.org. However, in order to store data for transfer of data between data acquisition system and computer, Open Ephys transfers the raw signal over USB (Universal serial bus) link, although work is under development to transfer data over PCI (Peripheral Component Interconnect) express. PCI Express has the advantages of lower latency and impressive transfer speeds. Compared to Ethernet (Metcalfe and Boggs 1976), which we are using for transferring data in the present study, PCI Express has slightly lower latencies, but is less developed and proven as a technique; therefore we have chosen Ethernet. Future development may yield PCI Express as a viable option also for our architecture, especially since it could be used to communicate directly with GPUs (Graphics Processing Unit) and FPGAs (Field Programmable Gate Array) (Bittner and Ruf 2013). Commercial acquisition systems such as Neuralynx and Plexon also have online transfer of data, but the data format for the online transfer is not open, although software exists for accessing and interacting with the data stream. For all current initiatives the data stream is limited to the number of channels in a single system, typically 512 or 1024 channels.

The aim of this study was to develop a system architecture and data encoding that enables handling of electrophysiological data from very large numbers of neurons (1 million or more) in real time (< 25 ms) over long periods of time.

## Method

### The System Architecture

**Fig. 1:**
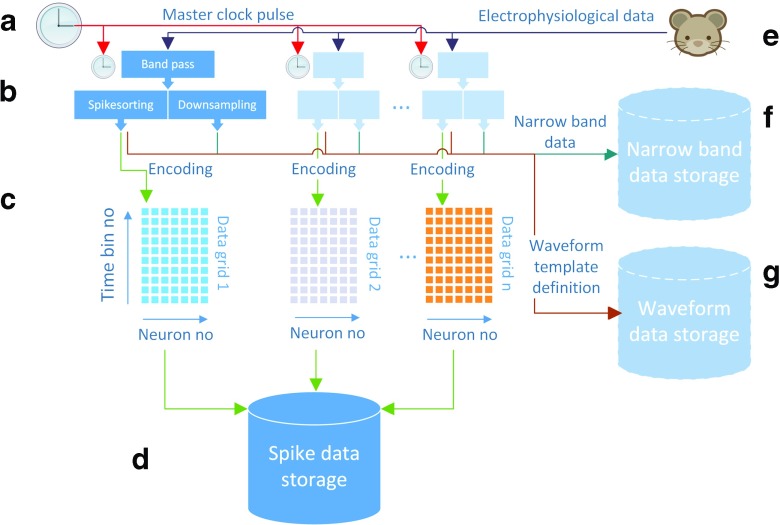
A proposed system architecture overview of storage for large amounts of real time data Arrows indicate direction and path of data flow. A master clock pulse (**a**) synchronizes *n* acquisition systems (**b**), which handles band pass filtering, spike sorting (for spike data) and down-sampling (for narrow band data), receiving electrophysiological data from subject (**e**). Neuronal spike data is encoded in a data grid of neurons * time bins (**c**). The resulting data grid is serialized and sent over to spike data storage (**d**), as well as to narrow band (**f**) and waveform data storage (**g**). In this work, **a** and **b** are simulated, **c** and **d** are implemented, while **f** and **g** are suggested not yet implemented components

In order to achieve the aim of this study, we suggest an architecture composed of the following parts (see Fig. 1):

*Master clock pulse (a*): - a pulse generator with the same frequency as the acquisition systems sampling frequency, synchronizing them through a clock input to the respective systems and providing a common timebase for all recorded data. Sub-microsecond precision is needed and is possible using an implementation IEEE 1588 precision time protocol (Correll et al. 2005)
*Acquisition systems (b)*: for storing electrophysiological data are optimized for reading a limited number of channels with high fidelity. Furthermore, we assume (as not all systems may allow for example spike template definition during an experiment), that they do the following preprocessing of data: 1) band pass filtering 2) spike detection 3) spike template generation, usually by clustering in a feature space 4) classification of new spikes into any of the clusters. Some systems may also perform online clustering and tracking, that is, adaptively changing cluster definitions during recording. In this work, we assume that electrodes and spike sorting software of sufficient quality to only generate single units and no multi units. Multi units may still be recorded by our suggested architecture, but the data format, as explained below, does not allow for spikes closer in time than 1 ms. As an experiment in the future can be expected to contain data from millions of neurons simultaneously, a recording of a single subject may be composed of many acquisition systems; with today’s processing power of a single system, an acquisition system is likely to be composed of maximally around 1000 channels (commercial systems such as Neuralynx, Plexon and Blackrock Microsystems all have a maximum configuration of 512 channels, while TDT have 1024) and are not designed to scale above this number of channels. The output of such a system is thus:
  - The spike times, sorted per neuron - This is the main input data into the *Encoder* (see below)
  - Narrow-band data (usually 1-300 Hz, down-sampled to 600 Hz, but could be set at other frequencies as well), recording Local Field Potentials (LFPs). This data is sent to the *Narrow-band storage*
  - The waveform characterizations that are deemed to come from a single cell, e.g. templates of waveform shape or similar descriptors. Waveforms characterizations (typically 32 data-points per waveform, assuming 32 kHz sampling rate), are periodically sent to the *Waveform storage*.
  It should be noted that a massive multi channel recording must consist of many different acquisition systems, which each have an array of electrodes for a specific part of the central nervous system of the subject, since a processor (at least with current computational power) only can handle a limited number of operations during a certain time, for example online spike sorting. As the acquisition systems are running in parallel, they gather data independently as they do not share a common memory area. In our suggested system architecture, they are integrated in the *spike data storage* and the *waveform storage*, permitting extremely fast analysis of spatiotemporal spike patterns.
*Encoder (c)*: - composed of in-house developed software providing a real time interface for the acquisition systems to an encoded format for the spike data, which is described in detail below.
*Spike data storage (d)*: - After transmission of data over network, the spike data storage, a custom built software written in Python, receives the data from the acquisition system encoder/adapter and then writes and integrates it into the common data storage. Also the storage buffers notifies listening applications that data is available for analysis and/or visualization.
*Narrow-band storage (f)*: - Since Local Field Potentials (LFP) may carry essential information, we foresee the use of storage for defined narrow bands, for example low frequency bands, in future extensions of the architecture.
*Waveform storage (g)*: - Since the spike waveforms are essential in order to evaluate cell identity (Bar-Hillel et al. 2006; Fraser and Schwartz 2012), changes in their features have to be tracked over time. For this purpose, we also propose a secondary storage for the waveforms that only records a subset of the neurons at a given time and cycles through all the neurons periodically in order to track the waveforms shape progression of all neurons. The waveform storage may reside on the same computer as the spike data storage, since it does not require such extensive bandwidth as the spike data storage, and for practical rapid access if using waveforms in analysis calculations.

Of these parts, only the *encoder* and the *spike data storage* are currently implemented in our architecture, with the acquisition systems being simulated.

As a new session is started, the project parameters, e.g. electrode type and number of neurons are fetched from the project server database, a MySQL database server. If needed, session specific meta data is entered, for example the stimulus type of the specific session. The controller application then starts up the various system components. Once the session is established and data is acquired, the data flows from the acquisition system(s) through the system components to the data storages, as indicated in Fig. 1.

### Software and Data Format

Our aim was to develop a system architecture that enables handling of electrophysiological data from large numbers of neurons in real time over long periods of time. When using BMI in applications of read-out from the nervous system for control of a device, and/or stimulation for control of the nervous system, computations need to be done within a certain time limit in order to be relevant in relation to nervous system state. In general, the *real time* capacity of a system refers to its capability of responding within the specified time limit. Here we define this time limit as 25 ms (Markram et al. 1997). In order to achieve such short response times lots of information needs to be processed in parallel. This can be achieved by using different processors or cores in a computer or by time slicing of processor/core execution time. A limiting factor in this context is the size of the smallest set of instructions that may be run separately. Such a ‘smallest set’ is termed a thread of execution. A single-threaded solution will result in blocking as it will run on a single processor, handling many sources of data serially instead of in parallel, and thus blocking communication with other acquisition systems when serving one. Multi-threaded design, on the other hand, allows each thread to receive data from its corresponding acquisition system (and set of neurons) and execute independently.

MPI, Message Passing Interface (Gabriel et al. 2004), is a commonly used system which enables multithreading and also defining a communication mechanism (message passing) through a common memory area, which is needed when performing parallel computing in which the results are dependent on each other, which is the case in our application. By choosing MPI, we overcome the limitations of threading of the programming language, as this is abstracted away from the program implementation. For example, in Python the so called global interpretation lock (https://docs.python.org/3/c-api/init.html) stops true multi-threading in pure Python, but as each Python runtime environment is run in a separate MPI thread, this is bypassed. MPI also works together with HDF5 to support multi-parallel execution when writing and accessing data from the data storage (https://support.hdfgroup.org/HDF5/PHDF5). Using HDF5 together with MPI thus supports our aim to integrate and store neuronal data from distributed systems.

At the software level, Python has a wide range of available libraries, most importantly Scipy and NumPy (van der Walt et al. 2011) for handling data arrays, mpi4Py (Dalcin et al. 2008) which provides a Python API (Application Programming Interface) to MPI as well as h5py which provides a Python API to HDF5, but also an increasing number of neuroscientific libraries such as OpenElectrophy (Garcia and Fourcaud-Trocmé 2009), Neo (Garcia et al. 2014) and NeuraPy https://github.com/kghose/neurapy. Thus, Python is a suitable choice for a more complex software project. As an interpreted programming language, performance is however slow for computationally heavy tasks. This might however be addressed by precompiling critical parts, which in turn maps efficiently to machine code instructions. We have used Cython (Behnel et al. 2011) for this purpose for the parts of our system that require extensive computations.

### Software Implementation

**Fig. 2:**
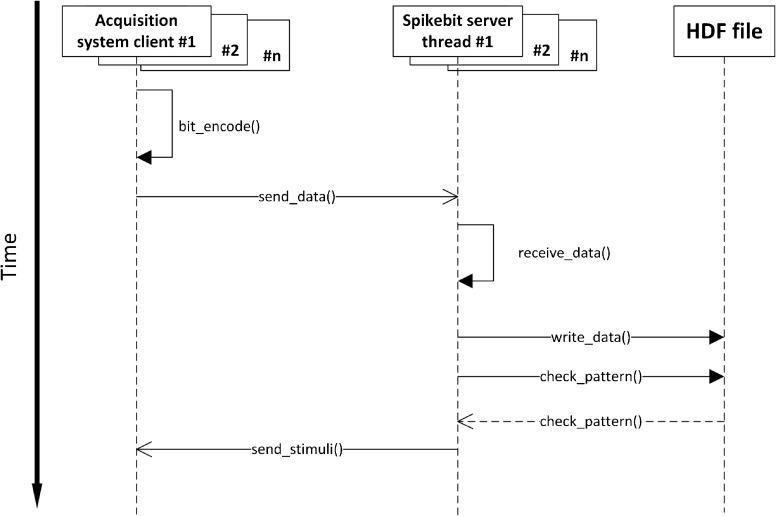
UML (Unified Modeling Language, Rumbaugh et al. (2004)) sequence diagram showing high-level overview of message exchange between acquisition system clients and server threads. Arrows correspond to function call in executing thread and/or message sent to other simultaneous threads on the same system. Multiple clients are communicating with multiple threads on the server side. The function/method arguments as well as the multiple threads function calls have been omitted for clarity


As the experiment starts, the system sets up project parameters, such as number of acquisition systems and their neuron count, through a user interface (either human interaction or through a REST-ful API, (Representational State Transfer) (Fielding and Taylor 2002). For each acquisition system, a thread is started using MPI. As recording starts, data is sent from acquisition system to data storage through TCP (Transmission Control Protocol), in our case over a high-speed LAN (Local Area Network), to which acquisition systems and data storage are connected. Before the experiment starts all parameters have been defined when configuring the project and the recording session parameters, for example the number of acquisition systems, the number of neurons in all acquisition systems, the acquisition system network ID, etc. The synchronized clock signal of the acquisition systems works together with the data format to ensure timing; the place in the data ‘matrix’ constitutes also a binning in time. Assuming that acquisition systems handle the clock pulse correctly, only checkup for data length and data sequence is needed, as TCP handles low level checks of the transmission. As the expected data length is known once the recording session is started and parameters are set for data size, the real time data transfer can thus be minimalistic; each received message is checked if it has the expected number of rows and columns. TCP handles that the messages are received in order. The storage system then receives the defined number of 32-bit integers for each set of 32 neurons and assigns it to the corresponding place in the HDF5 file. It is then possible to check for a pattern occurring at a previous instance in time by specifying which time slot in the HDF5 file to compare to. In Fig. 2, an overview of the messaging between the program execution threads of the clients and server is shown. Below follows sample Python code for the key operations, the encoding and transmission over the network (writing and reading from HDF5 file omitted, please see GitHub repository at https://www.github.com/NRC-lund/spikebit.git for full code). Also, the calls of routines have been omitted for readability and are instead visible in Fig. 2 together with some programming declarations such as type conversions. Some of the parts below are precompiled using Cython to C for speed in the actual application. In the first sample, Client thread - encoder, we show how the encoding is done in practice by using sparse matrix representation of the spike train and the unit IDs to perform a transformation into bit encoding. In the second sample, Client thread - sending data, we show how the data is sent by using the numpy internal data buffer representation to direct transfer from numpy to binary format, while we in the third sample, Server thread - receiving data, show how the data is received into a numpy array using the same internal buffer to fill up the array from the binary data received, and then directly writing the data to the HDF5 file.

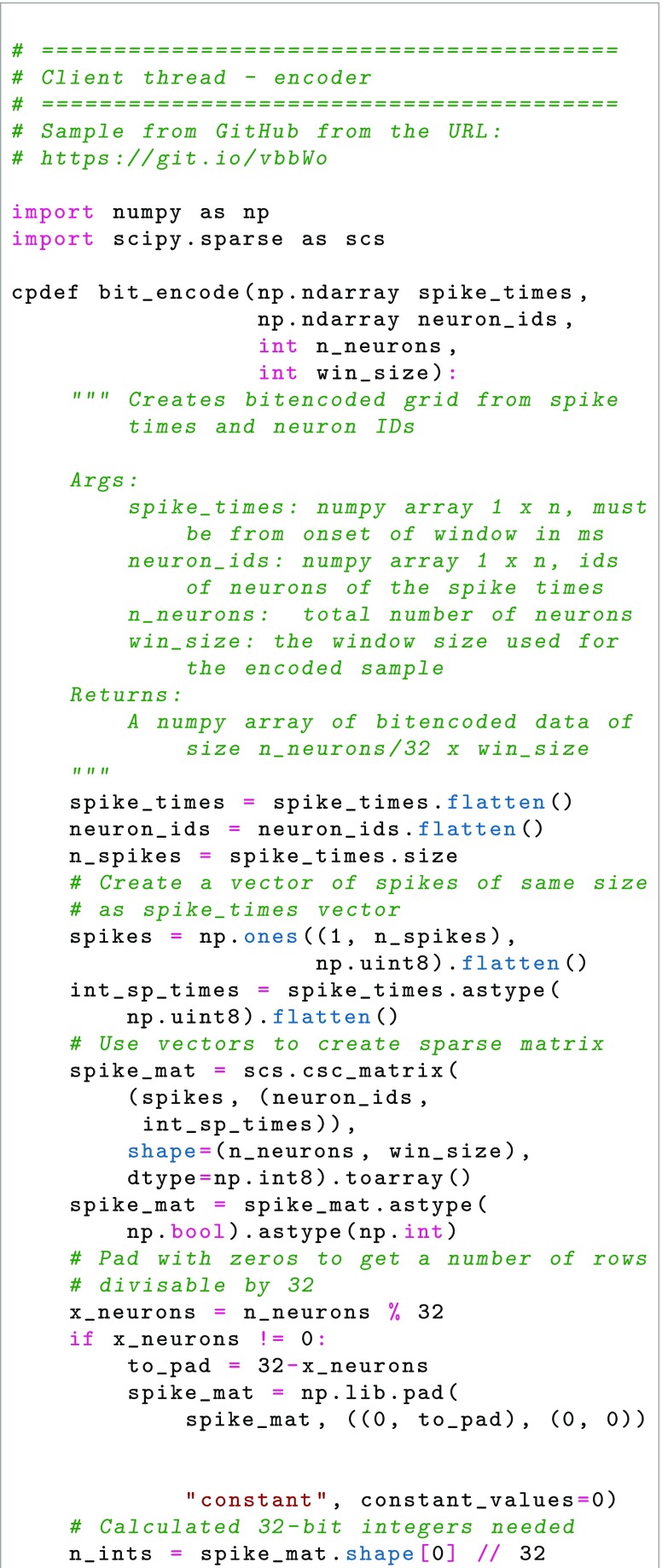
 
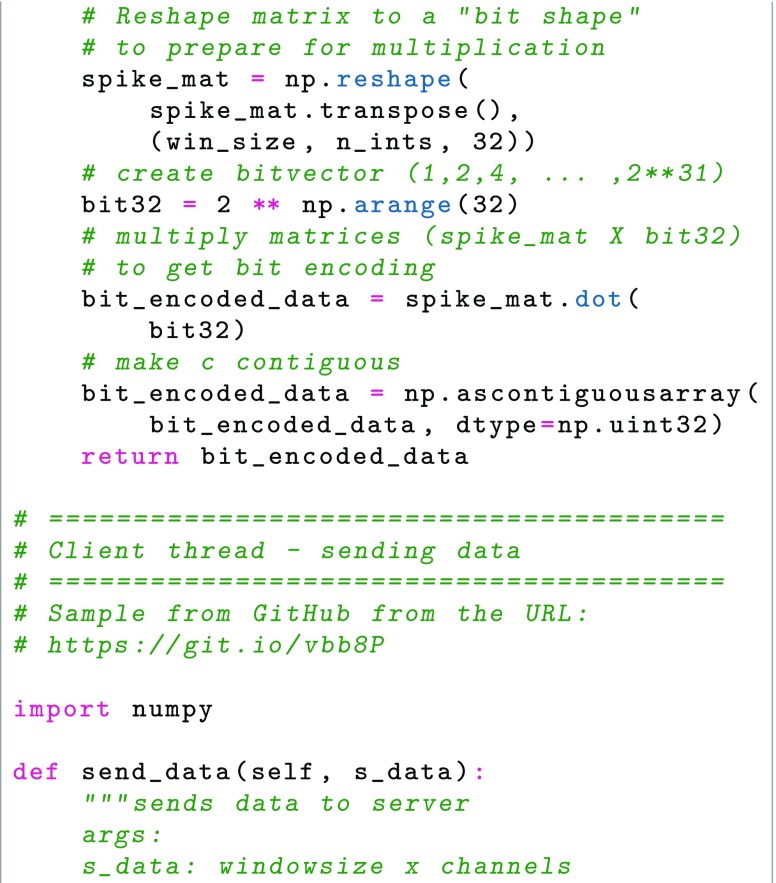
 
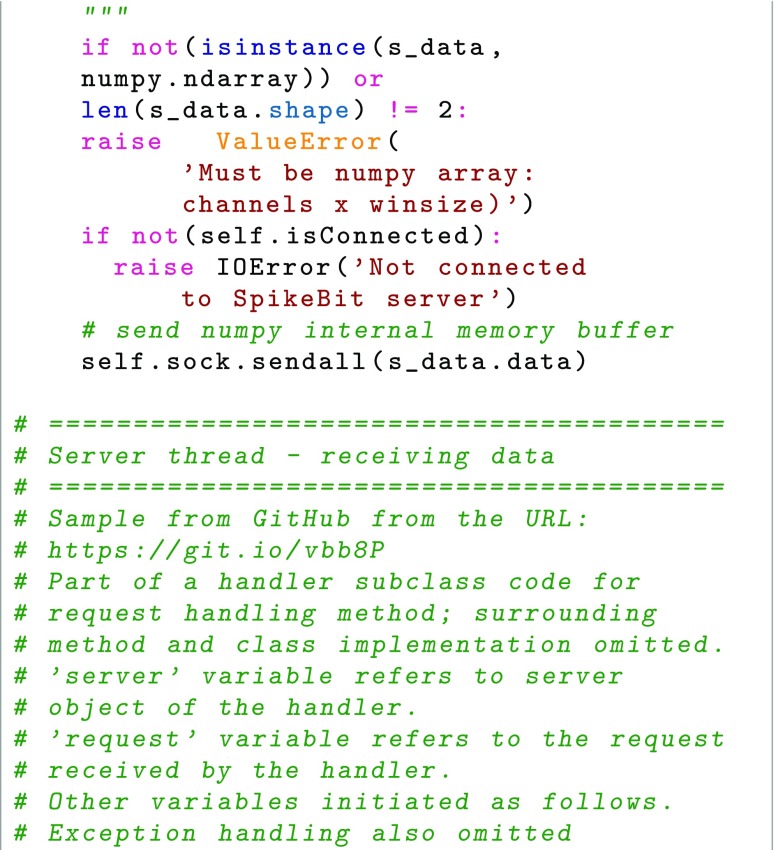
 
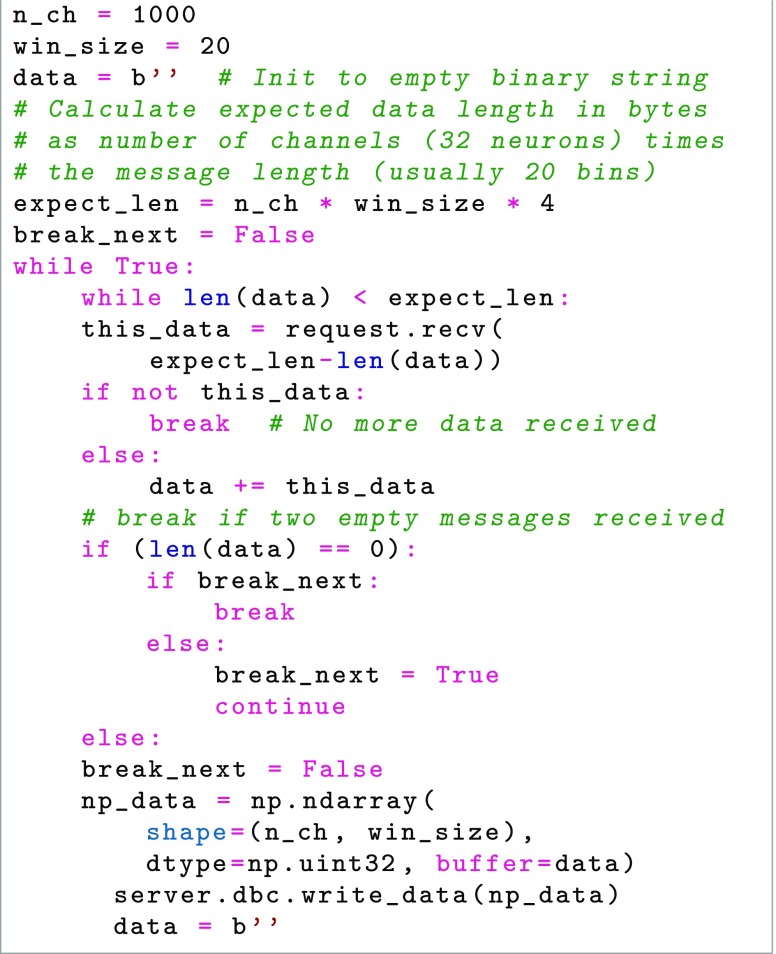


In order to evaluate potential for reduced data size during transfer, we tested compressing data using lzf- gzip and szip compression. These compression techniques reduced the data approximately to, respectively, 75%, 60% and 50% of original data size, but increased time to store data 6, 30 and 13 times, respectively. Since real time requirements in our case were deemed more important than data storage size, we did not use any other compression technique than our own bit-encoding in neither transfer nor storage of data.

To keep integrity of data from different datasets, we used parallel HDF5 and MPI together, as mentioned previously, allowing parallel processes communicating and writing to HDF5 file using MPI. File integrity is preserved as the communicating processes are not writing to the same dataset, but instead to different datasets, which are separately filled with data. If data is not available from one of the acquisition systems, this is detected as the expected sequence number in the header of the received message is erroneous. The missing data points are then filled with zeros, corresponding to no spikes received. Wide band data storage is handled by each of the connected acquisition systems. It will therefore be possible to perform offline analysis of wideband data together with the data in the integrated spike storage of our system in order to check integrity of the data.

### Encoding Algorithm and Spike Data Storage

As data rate transfers in the architecture will be limited due to hardware and software constraints, it is crucial to use data size as efficiently as possible, without trading speed. For this purpose, we developed an encoding algorithm and data storage format, which reduces data size to a minimal format needed for rapid integration, analysis and interaction with real time data.

**Fig. 3:**
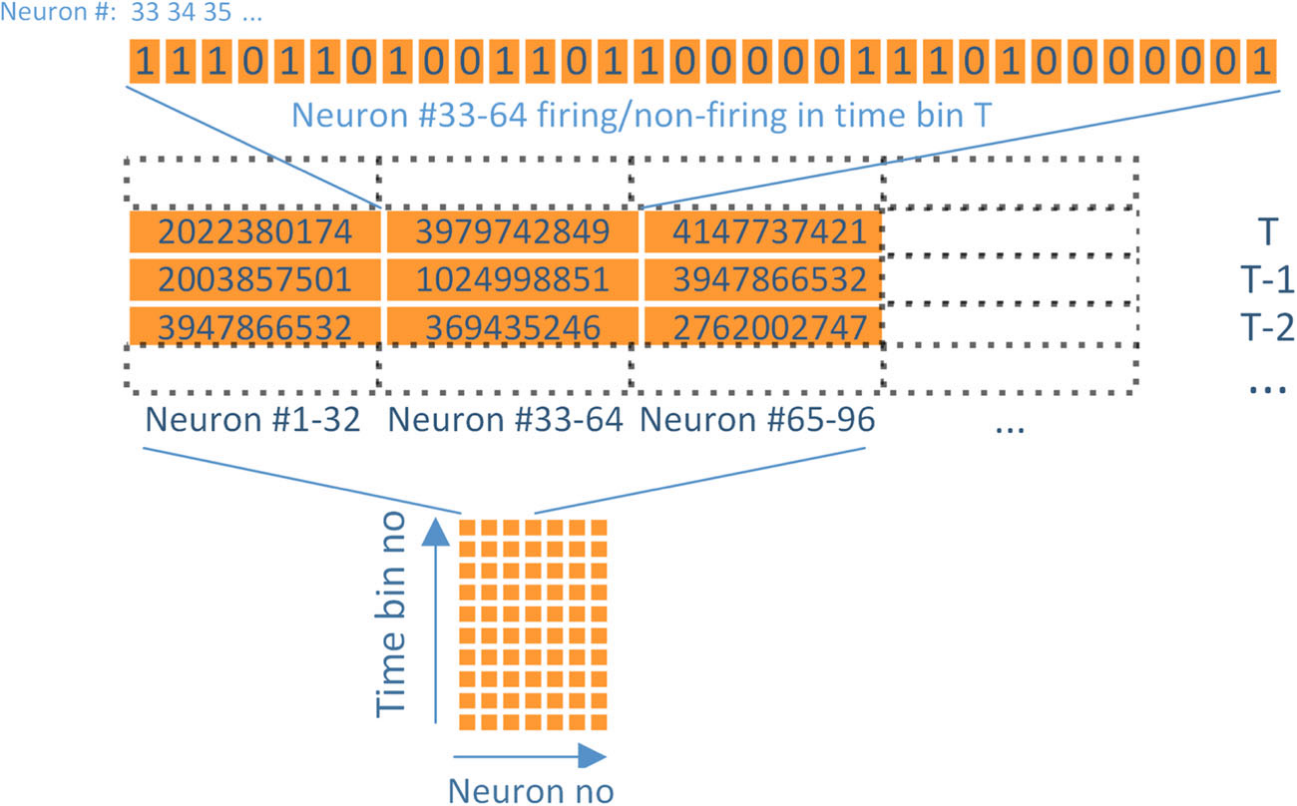
Proposed encoding of spike data in 32-bit integer data grids At any given time, each neuron spike/non-spike is encoded as a binary bit (1 or 0) in the corresponding time bin. The binary bits of groups of 32 neurons are then expressed as a 32-bit integer. The 32-bit integers are then grouped together in a data grid, where each row corresponds to a specific time bin and each column to the activity in a group of 32 neurons

Each spike is considered as a binary event in discrete time, either spiking during that period of time or not, which thus may be encoded as a single bit during a specific time as part of a 32-bit integer (64 bit could also be used if so desired). As for the time resolution, we assume that no neuron will fire with a frequency greater than 1000 Hz. The result is an array of 32-bit integers, of length N/32, where N is the number of neurons recorded in total at the time, see Fig. 3.

The input to our system is spike train data, that is waveforms, and time of the threshold crossing (or other time of detection, if for example a wavelet-based detection of spikes was used). If recording a large numbers of neurons, say up to 1 million, different systems are encoding a smaller part of the total number of neurons, although they should share a common timebase. The algorithm is then as follows:

1. Divide the neurons in segments of 32, each corresponding to a bit in the 32-bit integer to be encoded. If not enough neurons exist for a segment, the reserve bits are set to 0. If new neurons are detected, the reserve bits are allocated in order. A finite number of segments (N/32) are pre-allocated in memory and file system cache, covering the maximum number of neurons expected to be recorded during the session in each acquisition system. If a segment is filled, a new bit segment from that acquisition systems segments is allocated. Neurons which are not recognized to have been identified before will always get “new” IDs. We did not reuse IDs, but rather created a new bit group in order to store this information.
2. For each neuron, if a spike was detected since last transfer, the bit corresponding to that neuron is set to 1, otherwise it is set to 0. The result is a 32-bit integer.
3. Each integer in its position of the array to be sent is set to the resulting value, yielding a N/32 length array.
4. The array, which now encodes whether the neurons recorded by the acquisition system fired or not during the latest millisecond, is sent to data storage (HDF5 file in our system) for concatenation with previously recorded arrays and arrays from the other acquisition systems.

This encoding results in a data format, in form of a *m x n* size matrix, with *m* times sampled, in 1 ms bins, and n * 32 neurons. A recording of 15 minutes of 1 000 000 neurons would thus result in a *900 000 x 31 250* matrix, which if stored non-compressed would be of a size of 105 GB when using an HDF5 file to store the data. The software architecture is capable of recording longer periods of time; the size of the file system is the limit as HDF5 as data format has no limit in file size.

### Scalability Evaluation

Scalability refers to how well a system handles increasing load. We supervised maximum sampling rate and time to feedback for different configurations in order to evaluate the scalability for our system architecture. The assumption for the evaluation is however that the architecture is provided input from synchronized (through the clock signal) acquisition systems, each handling in the range of thousands of neurons each. We have tested the following scaling principles:

- *Neuron scaling:* Maximum sample rate for an increasing number of neurons from a single acquisition system without losing data. **Test success criterion:** The sampling rate stays above 1000 Hz sampling rate per neuron, which corresponds to the maximum expected neuronal firing rate.
- *Acquisition system scaling:* Maximum sample rate for an increasing number of systems integrated in real time into the same data storage without losing data. Number of neurons fixed at 320 000 neurons per system. **Test success criterion:** The sampling rated stays above 1000 Hz sampling rate per neuron, which corresponds to the maximum expected neuronal firing rate.
- *Time to feedback:* The time from that the data is acquired, analyzed (using a basic threshold detection algorithm, calculating a moving average of the data), and eventually sent back to a stimulator (if a specific pattern was detected). **Test success criterion:** Time to feedback stays below 25 ms.

To test these parameters, we built a software test suite emulating an acquisition system in Python (with critical parts precompiled in Cython), which allowed for varying the number of neurons, as well as running parallel sessions, simulating additional systems (in our tests with a fixed number of neurons, 320 000), which all were simulated by a single computer for sending over a local area network, or the same computer as the data were stored on. The test suite did not however limit the sampling rate to the typically expected of 1000 Hz per neuron, but rather sent data as quickly as possible. If the system indeed was able to handle at least 1000 Hz, it was considered to be successfully able to store the data. No “new” neurons were created during the testing. Spike trains were generated to correspond to an encoded array of integers in sequence (e.g. array of 1001 at time T, array of 1002 at time T + 1, array of 1003 at time T + 2, etc), interrupted by events corresponding to firing simultaneously (array of integer (2^32^ − 1) = 4294967295). This pattern of spike trains is not likely to be observed physiologically, but is a demonstration of the capability of the system to detect a firing pattern.

Data were sent in two different ways: 1) directly over a 10 Gbit Ethernet wired connection or 2) through a local loop-back in the case where the simulator resided on the same computer as the storage. In order to validate that the system stored all generated data, the software test suite generated a predefined spiking stream, consisting of consecutive bit-encoded integers. The time to feedback was calculated as the time after which the simulator had generated a data stream that should be detected by the pattern analysis (typically a logical comparison of a signal mask and the most recently recorded array of unit firing patterns) and sent it to the stimulator.

For the varied number of neurons, a total of 1000 data messages of *n x 20* (corresponding to the 20 ms window) were sent over, where n is the number of neurons in the trial, for which the speed was measured from the size of the data received and the time it took to transfer it. This was then repeated for 10 trials. For the increased number of systems, the same procedure was used, except for only running one trial, since the parallel systems themselves are generating an increased number of messages by each sending 1000 messages. From the acquired times statistics were calculated, pooling all samples for all trials for a specific number of neurons for the neuron scaling evaluation using MATLAB box plot. When scaling the number of acquisition system, we instead pooled samples from each of the different acquisition systems.

We also performed a longitudinal test of the software to record for a longer period of time. We allowed 4 systems, each having 100 000 neurons, thus in total 400 000 neurons, to write to the repository during 24 hours. The number of neurons was limited for practical reasons in terms of file system size, but could easily increased for larger file systems, also for longer periods of time.

Data were analyzed and stored using a dedicated HP Proliant HL350E Server with Ubuntu Linux 15.04, 16 GB RAM memory, dual Intel XEON Quad Core 2.4 GHz processors and a single HP Solid State Drive. All network transfers were done using a 10 GBit/s network (for the setup streaming from different acquisition systems). Data were generated by a HP z400 workstation with 12 GB Ram memory and dual Intel XEON Quad Core 2.4 Ghz processors. All involved hardware is thus quite ordinary commercial off the shelf, and may readily be utilized by different laboratories. The data from the longitudinal test was stored on a Samsung NVMe SSD 960 PRO Solid State Drive.

## Results

### Scaling Results

**Fig. 4:**
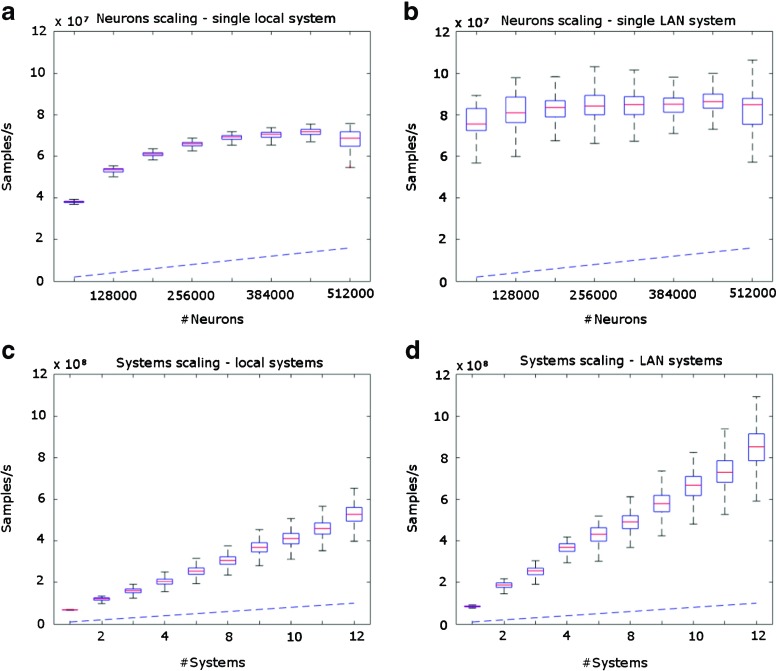
Box plots of throughput in samples/s as a function of number of neurons for a simulation with data **a** from a single system generating data locally, **b** from a single system sending data over 10 GbE LAN. Throughput in samples/s as a function of number of neurons **c** from an increasing number of systems generating data locally, **d** from an increasing number of systems sending data over 10 GbE LAN. For **c** and **d** the number of neurons were fixed at 320 000/system. Box plots are constructed with a 95% confidence interval (n = 1000). The blue dashed lines show the samples per second corresponding to the theoretical limit from the test success criteria with the sampling rate of 1000 Hz per neuron, which, as may be seen, the tested architecture is well above

Throughput for our proposed data format and software architecture was measured in samples/s, where a sample is defined as corresponding to a 32-bit integer, in which each bit is encoding the spiking of a neuron as defined earlier. It should be noted that performance is evaluated as maximal throughput, where the times between the spikes for a single cell during the simulations are less than 1 ms. Using this measure, neurons scaling stayed typically at a minimum level of 50 MSamples/s when running locally (using 95% confidence interval) for the localhost also when increasing number of neurons above 450 000 or more, see Fig. 4. The 10 GbE LAN connection performed more equally for different numbers of neurons, as could be expected since the local simulating process will be competing for resources on the same computer as the process writing to the database. As each bit has the capacity to encode a spike for a single neuron, as described above in the encoding section, 50 MSamples/s corresponds to 1,6 GSpikes/s. This limit is dependent on hardware, mainly network interface card, RAM memory and hard drive, but also on the length of the network buffer. For our simulations, we have used a network buffer of 20 samples (corresponding to 20 ms, assuming a sampling rate per neuron of 1000 Hz) for which the data corresponding of all different neurons is sent over together.

For the systems scaling, the limit was found to be the transfer of the network, or, if running locally, the speed of the memory, provided that large enough amount of RAM-memory is provided. Thus if not reaching up to either network or memory speed, a linear scaling pattern could be observed, limited by the number of parallel acquisition systems tested, in our case up to 10 acquisition systems. For 10 acquisition systems, we had a transfer rate of around 400 million samples per second when running locally (if using 95% confidence interval limit), which corresponds to a spiking frequency of 4 000 Hz (corresponding to the total number of samples for 320 000 neurons per acquisition system with 10 acquisition systems) per second, well over the test success criterion, which is 1000 Hz per neuron. When running over 10 GbE an increased performance was achieved with 600 million samples per second, which corresponds to spiking frequency of 6 000 Hz. The reason for this is the same as for the single system example, generating and writing data is competing for computational resources from the same CPUs. In both cases, the lower bound of the 95% confidence interval was also above the test success criterion of 1000 Hz per second.

The longitudinal test from 400 000 neurons resulted in a database (HDF5-file) with a size of 1014 GB (ca 1 TB). When checking for data integrity, it was confirmed that no data was missing.

### Analysis Results

The time to feedback stayed around 2.14 ms when including the threshold analysis operation. However, if the detected event is occurring early in a 20 ms window (i.e. the window time used, as described above), the detection of the event could be delayed a further maximum of 19ms. The result is, however, well below 25 ms, fulfilling the test criterion.

As already mentioned, the data format may be suitably sliced and concatenated with previous recordings, and is thus suitable for being sent over a TCP/IP network connection, or for local storage, as needed. As we outlined above, it may also be stored together with other previously recorded arrays as a multidimensional data storage of the firing pattern for all neurons of all acquisition systems. Performing pattern matching and recognition is very fast as the bit-wise comparisons are of low computational complexity and in Python for example, efficient and optimized software libraries exist for performing these operations. In our system, a pattern matching of data from one million neurons to a specific pattern over 10 time bins (10 ms) took around 0.5 ms.

## Discussion

As intracranial electrode technology develops, an increasing number of neurons may be recorded from. The ability to analyze and process the data in real time from this larger set of neurons must be considered a fundamental feature of future Brain Machine Interfaces. In this study, we develop and evaluate a scalable software architecture, with a defined interchange data format for mainly spike data which enables storage and analysis from at least a million of neurons simultaneously in real time.

The system architecture, including a bit-encoding data format and multi-threading storage software, has been tested for a maximum of 10 concurrent acquisition systems (acquisition systems recording from a specific set of neurons in the subject), each handling a subset of the recorded neurons, and the data rate is well over the required theoretical limit of 3.2 million neurons spiking each with 1000 Hz; the mean was 6 times higher when streaming over LAN and 4 higher when streaming from a local simulator program on the storage server. The system however has to be supported by databases which periodically record waveform data and verifies cell identity over time, as well as recordings of the narrow band signal as shown in Fig. 1. Regarding time to feedback, a feedback within 25 ms could be achieved. Increasing the buffer will lead to higher latencies, but having a shorter buffer will lead to inefficient transfer of data, as many small packets take longer time than a corresponding larger packet. This performance was reached on quite modest hardware and could probably be increased further using for example RAIDed hard drives and/or cluster of servers with each having a separate network connection. Furthermore, the proposed architecture allows for both online feedback back to the subject as well as online analysis of data. The present solution has been shown to be highly scalable, seemingly limited by only the transfer rate across the network, both when scaling up number of neurons and acquisition systems. The bit-encoding data format limits firing rate to 1 kHz maximal firing rate with a bin size of 1 ms. However, the bin size may be chosen smaller if desired to capture finer resolution, but the number of neurons that are able to be recorded will be lesser. For spikes from a single neuron, this is well below the minimum refractory period for most neurons. However, if data originates from unsorted MUA (Multi Unit Activity), our data format will not be able to capture multiple spikes within the bintime. We expect, however, that development of electrodes will result in improved signal to noise ratio and more precise and stable identification of single units, and some promising electrode designs already exist (Agorelius et al. 2015).

In databases of scientific data there has previously typically been a trade off between organization of data and performance in terms of data write and read rates for larger data sets. Relational data bases, at the very end of the spectrum with regard to organizational capabilities with extensive support for complex relationships between data, have been used by many research groups for storing scientific data, also recently for storing larger files such as images, movies and also electrophysiological data (Ljungquist et al. 2011; Ljungquist et al. 2016). However, if storing data values as single data entries in the data base, performance drops quickly, as INSERT statements in relational databases are costly (Schwartz et al. 2012). One way to go around this is to store item as so called BLOBs, binary large objects. However, performance drops with data sizes over a couple of MegaBytes in a BLOB (Stancu-Mara et al. 2014). It is also possible to DROP indexes during insertion, in order to increase performance slightly, but this would also result in problems during simultaneous INSERT and readout of data and during repeated INSERT statements. Relational databases in data intense real time applications is thus not feasible, although it might be fine for offline applications. In contrast, acquisition system developers have recently developed data formats which are prioritizing performance. For example the recent PL2 format by Plexon (http://www.plexon.com/pl2-file-system-overview) is capable of impressive data read and write speed. In Memory Data Grids (IMDG) is another technique which recently has received increasing attention in some real time intensive applications in other fields, such as finance (e.g. Hazelcast https://hazelcast.com and Grid-gain https://www.gridgain.com). These databases, while accepting impressive throughput in terms of transactions per second, however do not handle persistence and the requirement of being able to correlate the data to previously recorded data. Our suggested system architecture allows for multi-trial and multi-subject integration or cross-system integration within the same data source, combining the real time performance ability of instantaneous persistence together with capabilities of correlating the recently recorded data to a previously recorded pattern.

In previous studies of sensorimotor feedback loop learning (O’Doherty et al. 2011), bidirectional neural interfaces have been used in setups in which external world states decide which intracranial feedback to provide, as well as for studying and inducing spike-timing-dependent plasticity in corticospinal connections using an implanted chip for providing feedback (Nishimura et al. 2013). In the latter study, feedback within 25 ms was required, which is within the capabilities of our proposed system architecture, although with a small margin. If set too low, however, it affects data transfer performance. It furthermore enables trigging response trains also due to internal states, tracked and followed in real time by the database.

Our proposed architecture also allows for integration of distributed recording of many groups of channels, each handled by a separated processor or computer. In a neural interface with a large number of channels, this is required. Todays acquisition systems are in the maximum range of around 500 channels, which is around the limit of what a single computer can cope with. But distributing the computing power to possibly thousands of acquisition systems, which all are writing to the same data structure, allows for integration of these data sources in real time, which is a necessity for performing calculations concerning all of the channels of the neural interface. This allows for handling the massive amounts of data that may come from future hardware technology, which would allow for recording of many more neurons than today. When recording from these larger number of neurons, for performance reasons, it should be avoided to put preprocessing such as spike sorting and thresholding on the same processing device as the process handling the storage; thus we predict a distributed setup of smaller devices collaborating in a local network to store data into an integrated repository to be the future of neural interfaces.

In addition to integration, the suggested bit encoding results in reduced data size, for transfer and storage of data. If, for example, spikes would be encoded with timestamp and unit ID, which would occupy 32 bits each, they would need to be encoded with 64 bits per spike. Our suggested sampling rate of 1000 bit/s would then correspond to a maximum spiking rate per neuron of 1000/64 = 16 Hz. However, during a simultaneous discharge, e. g. so called neuronal avalanches, as seen in vitro, Beggs (2007) the neuronal firing rate may increase to many times this value, thus a system based on timestamp and unit ID for spike data would not be able to cope with the information. On the other hand, the bit format is currently more memory intensive as compared to representing spikes with unit ID and timestamp for firing rates below 16 Hz.

Another important benefit of the new bitencoded data format proposed in the present work is the capability for analyzing complex data patters during short periods of time. In closed loop experiments, latency must be kept minimal in real time interaction with data, as mentioned previously. This also includes analysis; in our test setup, we have implemented a basic pattern matching, which will return a similarity measure of the recorded pattern toward a desired pattern or previously recorded patterns. As the patterns, as well as the signals, are bit-encoded, these operations are very fast since whole arrays may be compared bit-wise simultaneously. In particular, it will enable binary neural network classifiers, which are deep neural networks constrained to operate on data and have weights that are binary (Courbariaux et al. 2016) to operate on the recorded data. This type of classifiers have recently attracted interest due to their significant lower computational and power cost in implementations, and have been shown to have both very high throughput (in the range of teraoperations per second) as well as very low latency (for most datasets in the microsecond range) in classifier implementations on specialized computational hardware such as FPGAs (Umuroglu et al. 2016; Fraser et al. 2017) in benchmarks of pattern recognition on established test data sets. In addition to impressive computational results, power consumption needed for calculations is comparably low (Umuroglu et al. 2016), which may result in future development of specialized analysis hardware in field conditions, e.g. carried or implanted electronics in the recording subject.

Further work involves implementation of the suggested architecture in a more extensive electrophysiological data analysis project, fully implementing also the suggested parts of the proposed architecture, for example the waveform storage. Also, the integration of multiple modalities, for example imaging, with electrophysiological data remains to be done. The work could be used together with existing methods for analysis of binned representations of massive parallel spike train data such as detection of synfire chain activity and sequences of synchronous events (Schrader et al. 2008; Torre et al. 2016).

Lastly, the suggested architecture enables storage of data from various subjects in real time in the same database, which opens up entirely new possibilities for analysis. Cross subject phenomena, for example electrophysiological recordings of social interactions between two subjects, could be investigated. This opens up for a range of new research that may be undertaken.

## Information Sharing Statement

All source code is freely available in a public repository at GitHub: https://www.github.com/NRC-Lund/spikebit.git.
